# Parental Role as Interpreter during Children’s Hospitalisation: Burden or Benefit?

**DOI:** 10.21315/mjms2024.31.1.20

**Published:** 2024-02-28

**Authors:** Yusrita Zolkefli

**Affiliations:** 1 PAPRSB Institute of Health Sciences, Universiti Brunei Darussalam, Brunei Darussalam; 2 School of Health in Social Science, the University of Edinburgh, Edinburgh, United Kingdom

Dear Editor,

I read the insightful article entitled ‘Understanding parental role in children’s participation in decision making during hospitalisation: An ethnographic study in Malaysia’ by Lee et al. (1) with considerable interest. While this study has identified three fundamental parental communication functions during children’s hospitalisation, for this commentary, I would like to focus on how parents have embraced the role of an interpreter. The goal is to shed light on a possible subtle imbalance of burden and benefit within the role.

As interpreters, parents are tasked with navigating the intricate information communicated, which can be a burden. It is not always easy when they must first make sense of the information before giving it to the children. While some parents may be willing to explain the jargon and technical terms, others may not feel comfortable doing so or have a limited understanding of the information (2). Moreover, when parents feel compelled to lie to their children to persuade them to comply with treatment plans, this is even more detrimental to their children’s trust in them. Children tend to assume the worst when kept in the dark, so it is preferable, to be honest from the outset (3).

However, a parent’s function as an interpreter can also be beneficial. As the authors have observed, children rely on their parents to manage communication with the nurse. I completely concur with this crucial point because, let’s face it, medical information is often complex and not always child-friendly. Most, if not all, parents may feel the dire need to be selective as they sift through the information for the child’s best interest. Thus, this allows parents to have a good strategy to avoid saying the wrong thing and overloading and overwhelming them with information. In other words, as reported in the study, the role helps parents translate information into a language children can comprehend, particularly when parents can speak their native language. Most importantly, the role has also provided the parents with a greater opportunity to advocate for their children, which includes asking questions, expressing concerns, and speaking up for the child (4).

Lee et al.’s study highlighted the parental role in promoting children’s decision-making participation. Although it seems almost natural to assign the parents the role of interpreter, as healthcare professionals, the role must be facilitated with prudence and sensitivity so as not to overburden and cause tension. Parents must be assisted in developing the confidence and competence they need to feel empowered to embrace the role (5). It would also be more reassuring if healthcare professionals could take the initiative to keep track of parental needs and offer appropriate support when the role becomes more challenging.
